# Non-enzymatic dual-mode plasmonic framework for robust bioanalyte detection

**DOI:** 10.1038/s41598-026-53629-7

**Published:** 2026-05-19

**Authors:** Olabisi Abdullahi Onifade, Mundzir Abdullah, Muhammad Hafiz Abu Bakar, Mohd Adzir Mahdi, Ahmad Shukri Muhammad Noor

**Affiliations:** 1 Institute of Nano Optoelectronics Research and Technology (INOR), Universiti Sains Malaysia, 11800 Penang, Malaysia; 2 Wireless and Photonics Research Center (WiPNET), Faculty of Engineering, Universiti Putra Malaysia (UPM), 43400 Serdang, Selangor Malaysia

**Keywords:** Dual-mode, Plasmonics, Gold nanoparticles, Reduced graphene oxide, Bioanalyte, Materials science, Nanoscience and technology, Optics and photonics

## Abstract

Quantitation of biological analytes at point-of-care remains challenging, particularly in complex media with competing species. Uric acid (UA), a clinically significant bioanalyte, is especially difficult to measure due to interference and the limited stability of conventional sensors. This work presents a non-enzymatic dual-mode plasmonic sensing strategy that integrates propagating surface plasmons with localized nanoparticle-driven resonances to enhance interaction strength and improve optical signal definition. The sensing interface features a multilayer nanostructure of gold film, APTES-modified gold nanoparticles, and reduced graphene oxide, providing reinforced light–matter interaction and selective surface affinity. The sensor achieves a high sensitivity of 0.2258°/(mg/dL), a low detection limit of 0.0446 mg/dL, and a high binding affinity of 1451.85 (mg/dL)⁻¹ across UA concentrations of 1–12 mg/dL. Selectivity studies show a pronounced resonance shift of 1.6645°, with interference suppressed to ~ 10% even in mixed solutions. Long-term performance assessments reveal less than 0.3% drift after 30 days, 97.2% sensitivity retention following 10 regeneration cycles, and stability above 90% maintained over 90 days at temperatures exceeding 25 °C. These results demonstrate a robust, regenerable, and interference-resistant platform suitable for real-time UA monitoring and adaptable to other clinically relevant bioanalytes.

## Introduction

The growing demand for rapid, accurate, and robust diagnostic tools has accelerated the development of high-performance sensors capable of real-time, point-of-care monitoring^1–3^. Such technologies are central to personalized healthcare, early disease detection, and continuous therapeutic assessment, where reliable quantification of biological analytes is essential. Among clinically significant biomarkers, uric acid (UA) is widely used to assess metabolic disorders, including gout, chronic kidney disease, and cardiovascular complications^4–6^. Accurate UA monitoring is therefore critical for timely diagnosis and treatment.

Conventional UA detection methods such as colorimetric assays, electrochemical sensing, uricase-based enzymatic methods, and high-performance liquid chromatography (HPLC), are well established but hindered by labour-intensive sample preparation, long assay times, and susceptibility to interference from competing analytes including D-cystine, glucose, urea, and creatinine^7–10^. Electrochemical sensors, typically employing amperometry or voltammetry on modified electrodes provide rapid point-of-care detection but suffer from overlapping oxidation potentials of interferents, necessitating extensive surface engineering and sample pretreatment^11–14^. Uricase-integrated electrochemical and optical sensors alike face enzyme degradation, with operational lifetimes often limited to days under physiological conditions (pH 7.4, 37 °C), curtailing continuous monitoring^15–18^. These conventional approaches underscore the need for non-enzymatic plasmonic platforms employing surface plasmon resonance (SPR) or localised surface plasmon resonance (LSPR) techniques, which leverage refractive index (RI) specificity over redox interference for robust anti-interferent performance.

SPR enables label-free, real-time detection with high RI sensitivity and versatile surface functionalization^19–21^. In conventional prism-coupled SPR configurations such as Kretschmann and Otto geometries, surface plasmons are excited at a planar metal–dielectric interface^22–25^. LSPR, in contrast, arises from the collective oscillation of conduction electrons in metallic nanostructures, producing highly confined electromagnetic fields that enhance surface-specific interactions^26,27^. Across both SPR and LSPR platforms, commonly employed interrogation modalities, including angular, intensity, phase, and wavelength interrogation, offer different trade-offs in sensitivity, resolution, and system complexity^28–30^. Despite their widespread use, conventional SPR systems are constrained by the finite penetration depth of the evanescent field, which can limit sensitivity toward low-concentration or weakly interacting analytes^31,32^. LSPR-based sensors, typically relying on plasmonic nanostructures such as gold nanoparticles (AuNPs), provide stronger near-field enhancement and improved local sensitivity^33–36^, however, they are often limited by narrow dynamic range, reduced angular resolution, and stability issues associated with nanoparticle aggregation or surface degradation^37,38^.

In plasmonic sensors, nanomaterial selection dictates key metrics like refractive index (RI) sensitivity, adsorption efficiency, field enhancement, and operational stability^39,40^. 2D materials such as graphene or reduced graphene oxide (rGO) boost performance through high carrier mobility, π–π stacking for analyte capture, and evanescent field extension, while sacrificing some sharpness in resonance dips^41,42^. 0D quantum dots (graphene quantum dots, carbon quantum dots) provide intense LSPR hotspots via quantum confinement and discrete packing with AuNPs, yielding superior local RI sensitivity but potential aggregation issues^43^. 1D nanostructures like Au nanorods or CNTs offer directional plasmon coupling for polarization-selective detection, whereas hybrid nanocomposites (e.g., MoS₂-AuNPs, MXenes) combine conductivity, defect-engineered active sites, and biocompatibility for broad analytes from biomolecules to gases^44,45^.

To overcome the inherent limitations of standalone SPR and LSPR systems, a dual-mode plasmonic platform was previously reported, integrating the extended field sensitivity of SPR with the localized enhancement of LSPR through a multilayer nanomatrix comprising gold thin films, APTES linkers, AuNPs, and graphene quantum dots (GQDs)^46^. This platform enabled label-free UA detection with improved sensitivity and selectivity across physiologically relevant concentration ranges^47^. However, key performance aspects, including long-term stability, binding kinetics, operational robustness, and comprehensive benchmarking were not fully explored. In the present study, these gaps are addressed through detailed physicochemical characterization and an expanded evaluation of performance metrics, including sensitivity, selectivity, limit of detection (LOD), binding affinity, and resistance to interfering species. Operational robustness is systematically assessed via 30-day baseline drift analysis, multicycle reusability tests, and controlled thermal and storage stability studies.

Building on this foundation, two primary advancements are introduced. First, reduced graphene oxide (rGO) replaces GQDs as the active 2D nanomaterial, offering improved sheet-like coverage, higher electrical conductivity, enhanced mechanical stability, and more cost-effective scalability^48,49^. Second, a more comprehensive long-term performance assessment is conducted, including thermal stability (4–30 °C) and extended storage stability (up to 90 days) under both ambient and refrigerated conditions. Although the core SPR–LSPR architecture remains unchanged, the incorporation of rGO leads to enhanced sensitivity, binding affinity, and selectivity. Collectively, these improvements, together with the expanded stability analysis, provide a more realistic and in-depth evaluation of the sensor’s durability, scalability, and practical applicability for continuous and point-of-care monitoring, while also offering deeper insight into the mechanisms governing dual-mode plasmonic sensing. The remaining part of this manuscript is organized as follows: “Materials and methods” section describes the materials, fabrication, and experimental procedures; “Results and discussion” section presents sensor characterization, performance results, and comparative analysis; and “Conclusion” section concludes with key findings and perspectives for next-generation plasmonic sensing.

## Materials and methods

Performance of the dual-mode plasmonic sensor for uric acid detection was evaluated through experiments involving sensor fabrication, bioanalyte preparation, and optical characterization. The design of the nanostructured interface drew on insights from previous SPR and LSPR studies, highlighting how gold, gold nanoparticles, graphene derivatives, and APTES linkers enhance sensitivity, binding efficiency, selectivity, detection limits, and operational stability under physiologically relevant conditions^18,46,50–52^.

### Chemicals and reagents

All reagents were analytical grade and used without further purification. Uric acid (UA, ≥ 99%), NaOH (97%), glucose (≥ 95% GC), D-cystine (98%), urea (99–100.5%), and creatinine (≥ 98%) were sourced from Sigma-Aldrich (USA). Deionized water (DI) was used for all solutions. Glass coverslips (22 × 22 × 0.13 mm), acetone (≥ 99%), hydrogen peroxide (30%), sulfuric acid (95–98%), and 99.99% gold targets were also obtained from Sigma-Aldrich for thin-film deposition. reduced graphene oxide (RGO; ACS Materials) and APTES (99%) were used to functionalize the sensing interface. AuNPs were synthesized from HAuCl₄·3 H₂O and trisodium citrate dihydrate using the citrate reduction method. All concentrations were prepared using standard dilution principles (C₁V₁ = C₂V₂).

### Preparation of the target bioanalyte and interferents

UA solutions with concentrations of 1, 3, 5, 7, 9, and 12 mg/dL were prepared by dissolving UA powder in DI water. A small amount of NaOH was added to facilitate complete dissolution of UA and maintain pH stability across the physiological range. The solutions were stirred at 300 rpm for 30 min using a magnetic stirrer to ensure complete homogeneity. Desired UA concentrations were obtained by appropriate dilution using the *C₁V₁ = C₂V₂* relationship. To assess selectivity, solutions of competing analytes that also function as physiological interferents—glucose, urea, d-cystine, and creatinine—were prepared at a concentration of 9 mg/dL under identical conditions. All solutions were stored in sealed containers at ambient temperature to ensure consistency during sensing experiments.

### Fabrication and deposition process of the dual-mode sensor nanomaterial

**Fig. 1:**
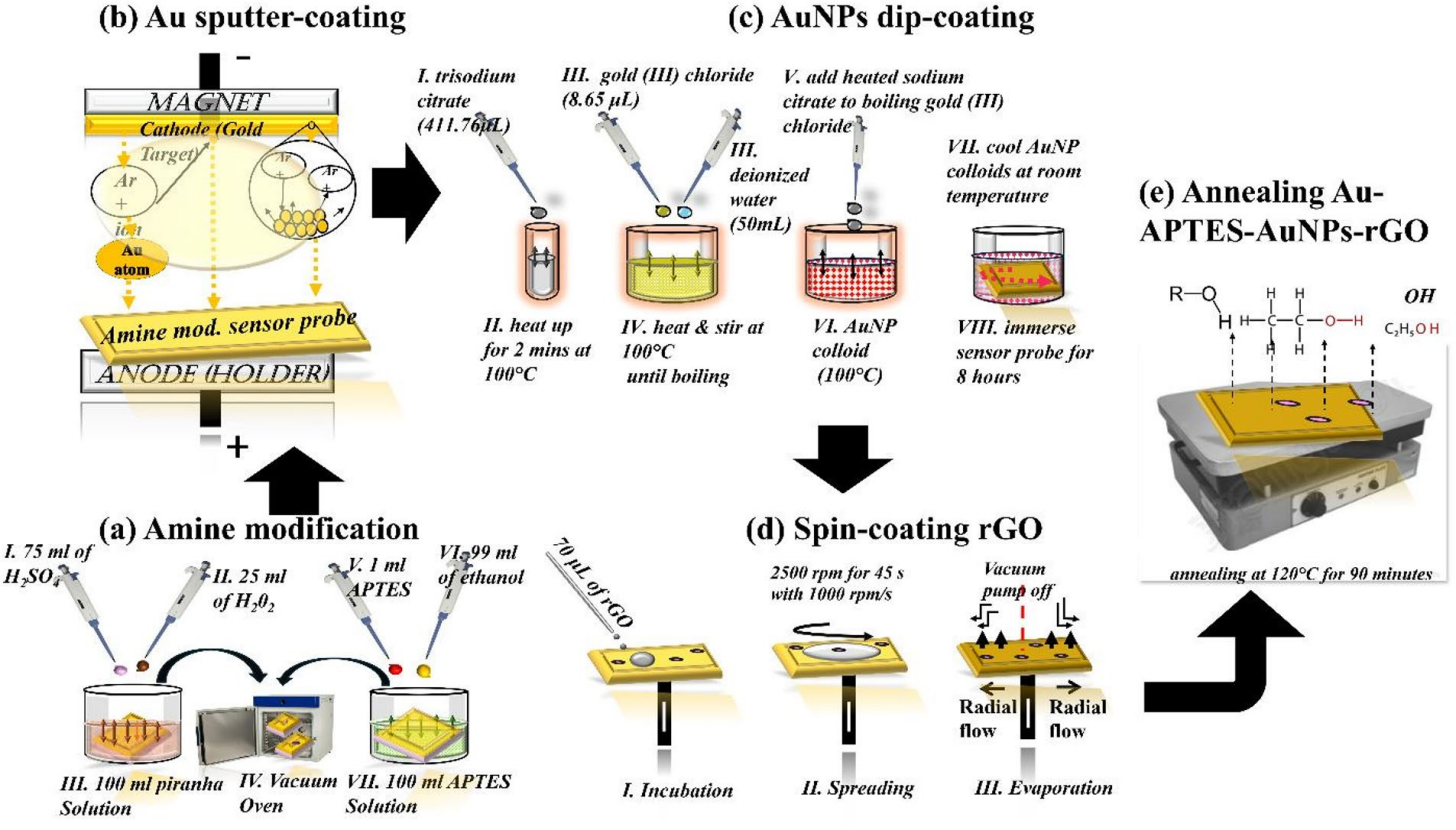
Fabrication and deposition process of dual-mode sensor.

Glass coverslips were cleaned in piranha solution (H₂SO₄:H₂O₂ = 3:1) for 30 min to remove organics and hydroxylate the surface. A 50 nm gold film was sputter-deposited (EMITECH K55X; deposition rate 0.746 nm/s, 30 mA sputter current, 130 mA clean current, 67 s). The gold surface was functionalized by immersing in 1% APTES–ethanol for 24 h, followed by ethanol sonication (5 min) and oven drying (30 min). Citrate-reduced AuNPs (~ 20 nm) were immobilized by 8-hour immersion and subsequently dried and annealed (20 min and 5 min, respectively). Reduced graphene oxide synthesized via a modified Hummers’ method for controlled graphite oxidation, followed by thermal reduction/exfoliation at 800–1000 °C under N₂/Ar, was deposited onto Au*–*APTES*–*AuNPs sensors using sheets^53,54^. The rGO was dispersed at 1 mg/mL in anhydrous ethanol and ultrasonicated (200 W, 20 kHz, 40% amplitude) for 45 min in an ice bath to prevent overheating^41^. Deposition was performed by spin-coating 70 µL of the suspension onto the 1 cm² sensing region at 2500 rpm for 45 s with 1000 rpm/s acceleration, producing a uniform monolayer, optimal for evanescent field penetration without quenching the SPR signal^42^. Thermal annealing at 120 °C for 90 min under removed residual hydroxyls and ethanol, restored π-conjugated sp² domains, and promoted van der Waals and covalent adhesion without sintering the AuNPs^48,55^. Loosely bound rGO was removed by triple rinsing with PBS (pH 7.4) and ethanol, followed by N₂ drying and storage in a desiccator. The schematic illustrating the complete fabrication and deposition process of the dual-mode sensor nanomaterial is shown in Fig. 1.

### Characterization and performance evaluation of the dual-mode sensor

The structural and optical properties of the dual-mode plasmonic interface were verified using HR-TEM (JEOL 2100 FETEM) to assess AuNPs morphology and size distribution, FESEM (JSM 7600 F) to evaluate nanomatrix uniformity and surface topography, FTIR (Bruker, USA) to establish chemical bonds as well as functional groups, and UV–Vis spectroscopy (Lambda 35) to confirm the plasmonic response. These characterizations ensured that the fabricated sensor met the intended design specifications. Comprehensive performance evaluation was then conducted, covering sensitivity, limit of detection, selectivity, binding affinity, and stability. Long-term, thermal, and storage stability were assessed under physiologically relevant conditions over 30 days. Together, these analyses establish the dual-mode sensor’s robustness and analytical reliability.

### Dual-mode plasmonic sensor measurement setup

**Fig. 2:**
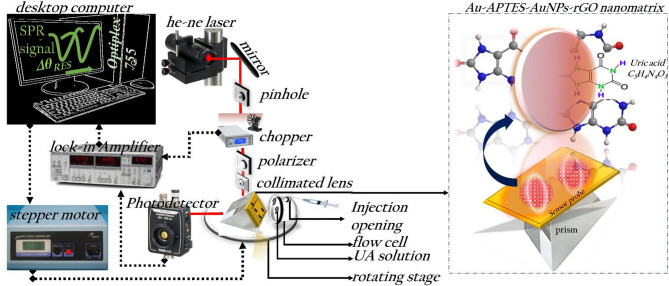
Dual-mode plasmonic sensor measurement setup.

The optical characterization was performed using a custom-built SPR platform, previously described in^51,52^. In brief, a He–Ne laser (632.8 nm, Thorlabs) was filtered, modulated via a mechanical chopper (SR 540), and polarized before being directed into an SF11 prism mounted on a motorized rotating stage (Newport MM 3000). The incident light excited surface plasmons on the nanostructured sensor surface, where molecular binding events altered the local refractive index and shifted the resonance angle. Reflected light intensity was recorded by a photodetector (Newport) and processed by a lock-in amplifier (SR 530) to enhance the signal-to-noise ratio. All components were controlled via a custom MATLAB-based interface, enabling real-time data acquisition, analysis, and storage. Figure 2 illustrates the experimental arrangement and signal detection workflow. All SPR measurements were conducted using *n* = 3 independently fabricated sensors, unless otherwise stated. Reported values represent the mean of three measurements, with error bars indicating the standard deviation (~ 1–10% RSD).

## Results and discussion

The dual-mode plasmonic sensor was carefully examined to understand how its structure, optical properties, and functional design influence UA detection. Each step—ranging from structural analysis and binding studies to sensitivity, selectivity, stability tests, and comparative benchmarking—was aimed at connecting the nanoscale architecture with overall sensor performance. These investigations reveal how combining SPR and LSPR with hybrid nanomaterials works synergistically, enhancing signal strength, analyte specificity, and long-term operational reliability.

### Dual-mode plasmonic sensor structural and surface morphology analysis

**Fig. 3:**
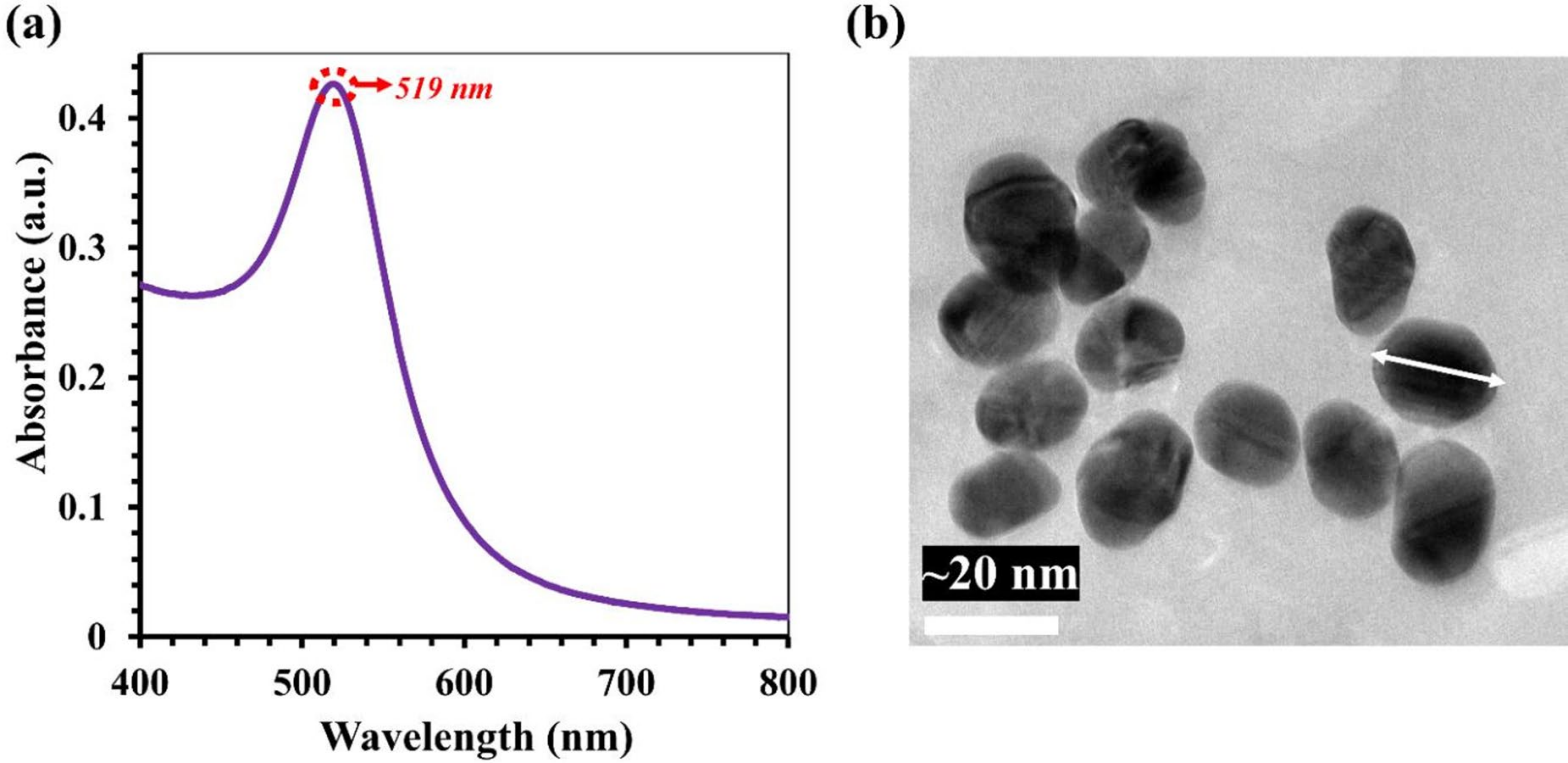
(**a**) UV–Vis absorption spectra of the dual-mode plasmonic sensor components, showing optical response of AuNPs, and (**b**) HR-TEM image showing AuNPs particle size.

The UV–Vis spectra in Fig. 3a reveal the optical characteristics of the AuNPs that form the functional nanointerface of the dual-mode plasmonic sensor. The AuNPs dispersion exhibits a distinct LSPR peak at 519 nm, consistent with quasi-spherical particles in the 10–20 nm range^56–59^. The sharp peak and absence of secondary features indicate minimal aggregation and a stable colloidal state. HR-TEM imaging in Fig. 3b verifies the monodispersed, crystalline nature of the AuNPs, showing clear lattice fringes, uniform morphology, and an average size of ~ 20 nm, in agreement with the UV–Vis results^60–64^. This monodispersity supports stable LSPR behavior and enhances near-field coupling.

**Fig. 4:**
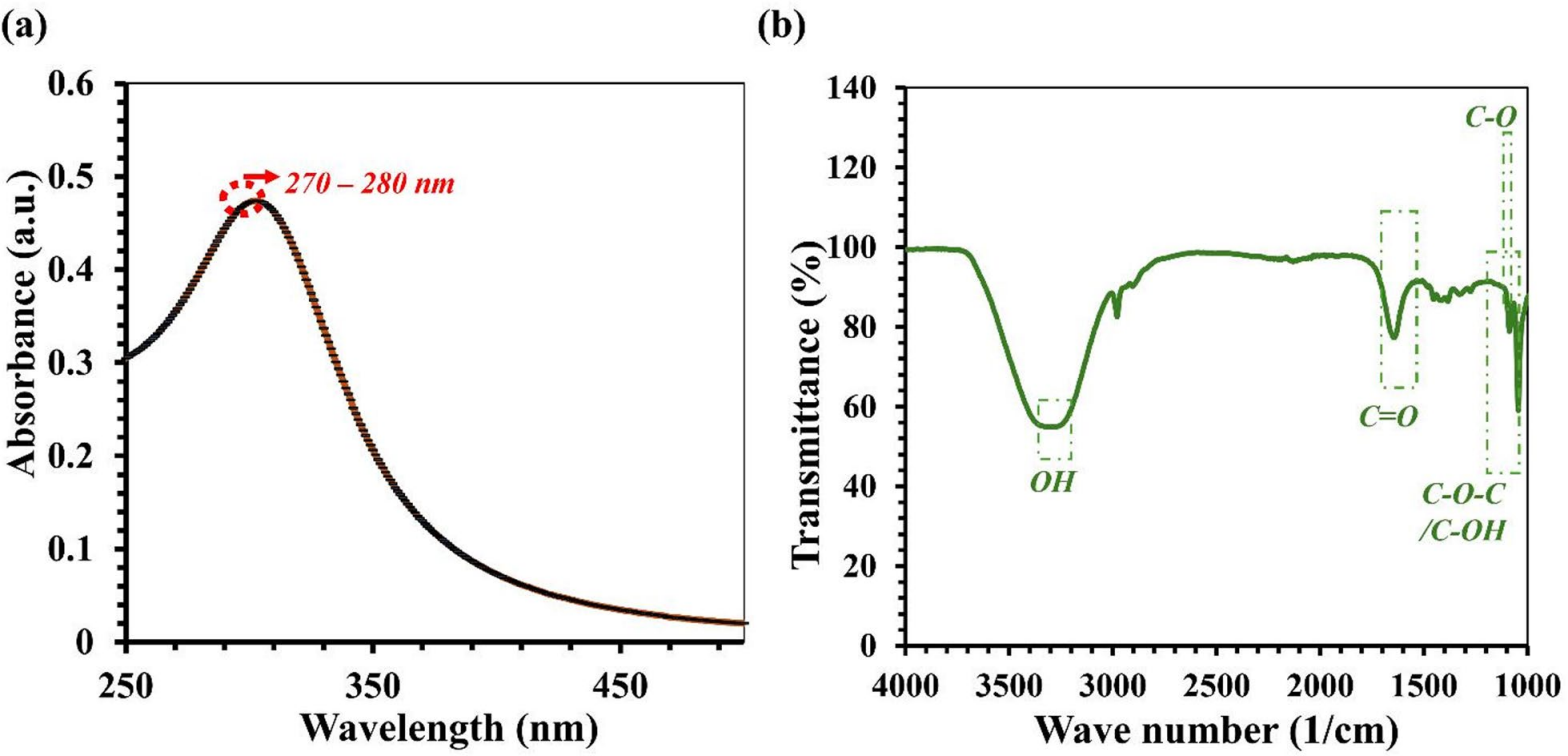
(**a**) UV–Vis absorption spectra of the dual-mode plasmonic sensor components, showing optical response of rGO, and (**b**) FTIR showing functional groups and chemical bonds present in rGO.

The rGO UV-Vis spectra (Fig. 4a) reveal a broad absorption maximum at 260–270 nm, signifying partial restoration of aromatic π-π* transitions in extended sp² carbon domains following graphene oxide reduction^65,66^. Beyond 300 nm, rGO displays a featureless, gradually declining tail into the visible-NIR (400–800 nm), attributed to light scattering by micrometer-scale sheets, sub-bandgap excitons, and free-carrier absorption akin to graphene’s linear dispersion^67–69^.

FTIR spectrum of rGO (Fig. 4b), acquired in ATR mode (4000 –500 cm⁻¹, 4 cm⁻¹ resolution), showcases dramatic suppression of oxygen-related vibrations—evident in the weak O–H stretch (~ 3400 cm⁻¹) and faint C=O (~ 1700 cm⁻¹)—signaling effective deoxygenation (C/O > 8–10) and sp² network restoration^70–72^. The dominant C=C skeletal vibration at ~ 1620 cm⁻¹ appears as a sharp transmittance minimum, with C–O (~ 1100 cm⁻¹) to C=C intensity ratio < 0.2 confirming > 85% oxygen removal and few-layer morphology suitable for sensor functionalization^68^. Notably absent are GO’s characteristic C-O-C/C-OH bands (1200-1000 cm⁻¹), while baseline stability across 3000 –1500 cm⁻¹ validates uniform preparation^71,72^. This graphitic profile correlates with conductivity gains (~ 50–200 S/cm films), essential for plasmonic field enhancement^72^.

**Fig. 5:**
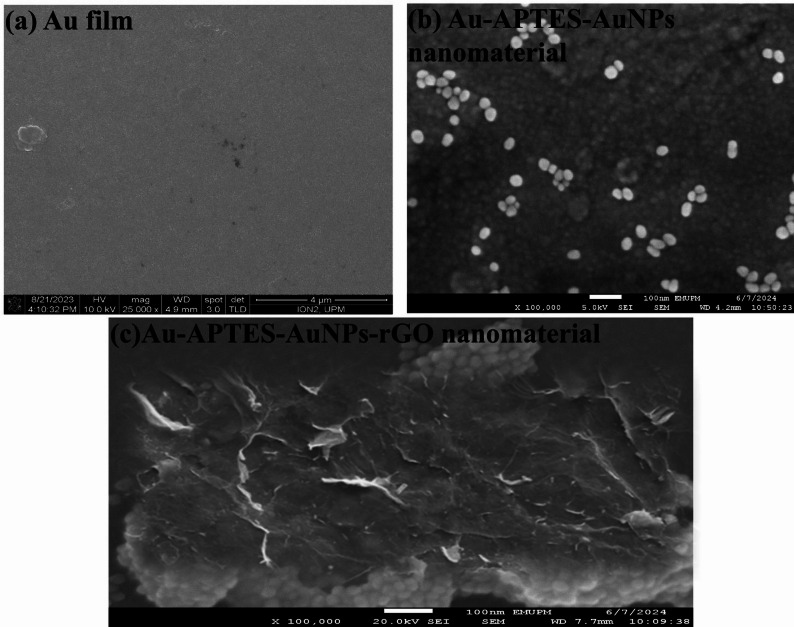
FESEM image showing surface morphology for (**a**) Au film, (**b**) Au*–*APTES*–*AuNPs nanomaterial and (**c**) Au*–*APTES*–*AuNPs*–*rGO nanomaterial.

The surface morphology of the sensor is shown in Fig. 5a–c. The sputtered gold film (Fig. 5a) forms a smooth, continuous ~ 50 nm layer with low roughness, ensuring efficient SPR excitation, reduced scattering losses, and a stable baseline signal^63,73–75^. Functionalization with AuNPs (Fig. 5b) produces a uniformly dispersed layer of ~ 20 nm nanoparticles that generate localized plasmonic hotspots without aggregation, thereby strengthening electromagnetic field confinement^76,77^. Deposition of rGO (Fig. 5c) results in thin, crumpled 2D sheet-like morphology wrapping and interconnecting the AuNPs, forming a hybrid Au–APTES–AuNPs–rGO nanocomposite. The characteristic wrinkled rGO topography increases effective surface area, provides π-conjugated pathways for enhanced conductivity, and creates additional nano-interfaces for analyte adsorption, contributing to superior sensitivity, faster response kinetics, and robust anti-interference performance^78,79^. Integrating the structural (UV–Vis, HR-TEM) and morphological (FESEM) analyses confirms the successful fabrication of a multi-layer plasmonic interface with coherent optical features and optimized surface architecture, enabling high-performance UA detection.

### Dual-mode plasmonic sensor binding analysis

The binding kinetics of the developed dual-mode sensor were analyzed using the Langmuir adsorption isotherm model calculated with Eq. (1)^50^,

1 $$\Delta {\theta _{RES}}=\Delta {\theta _{RE{S_{\hbox{max} }}}}\left( {\frac{{KC}}{{1+KC}}} \right)$$

where *∆θ*_*RES*_ is the resonance angle shift in degrees, $\Delta {\theta _{RE{S_{\hbox{max} }}}}$ is the maximum resonance angle shift at saturation, K is the binding affinity constant, and *C* is the UA concentration.

The Langmuir model assumes monolayer adsorption onto a homogeneous surface with equivalent binding sites, which is an idealization that does not fully capture the complexity of the Au‑APTES‑AuNPs‑rGO interface. The rGO sheet contains defects, edges, and oxygen‑containing functional groups, while the AuNPs surface may exhibit variations in local curvature and ligand density, leading to a heterogeneous distribution of binding energies^80,81^. Despite this, the Langmuir fit yields R² = 0.93, indicating that the model reasonably describes the overall trend of saturation‑limited binding^82^. Alternative models such as the Freundlich isotherm or multi‑site Langmuir approaches could account for surface heterogeneity and different binding affinities^83^, but they require additional fitting parameters and are not well‑constrained by the relatively narrow UA concentration range (1–12 mg/dL) used here. For the present work, the Langmuir model provides a simple, interpretable description of saturation‑limited adsorption and enables a straightforward comparison of binding affinity with prior SPR‑based platforms^50–52,84^.

**Fig. 6:**
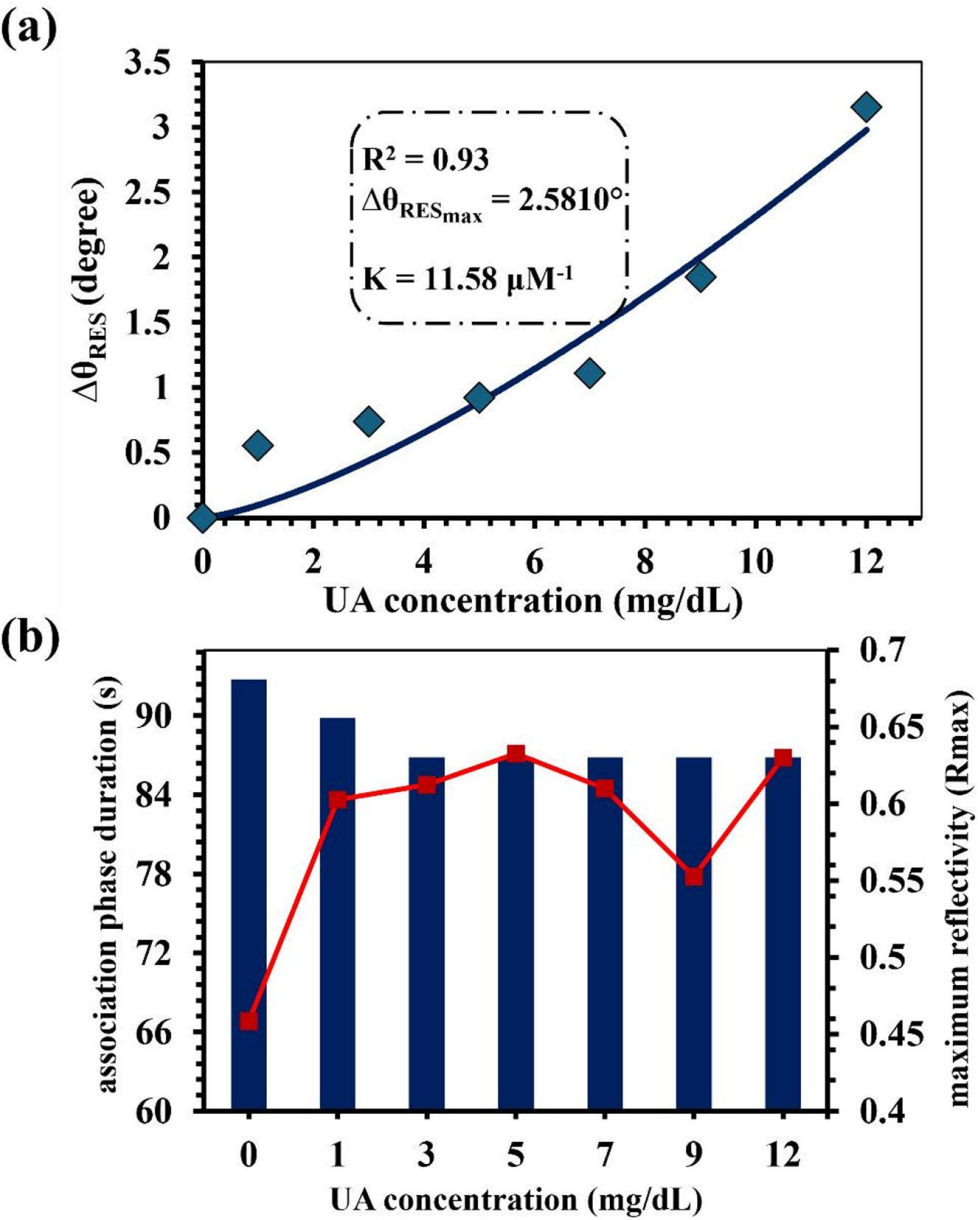
Dual-mode plasmonic sensor’s (**a**) binding kinetics, and (**b**) binding association.

Figure 6a shows a clear concentration-dependent increase in *∆θ*_*RES*_ over the UA range of 0–12 mg/dL. The nonlinear fit exhibits good agreement with the experimental data (R² = 0.93), yielding a $\Delta {\theta _{RE{S_{\hbox{max} }}}}$= 2.5810° and an affinity constant of = 11.58 M^−1^. These values indicate strong affinity between UA molecules and the functionalized plasmonic surface within physiologically and pathologically relevant concentration ranges. The gradual deviation from linearity at higher concentrations reflects progressive occupation of available binding sites and the onset of surface saturation, consistent with Langmuir-type adsorption behavior^52,85^.

To further examine the binding kinetics, the association phase of the reflectivity response was extracted and fitted using the exponential growth model defined in Eq. (2)^86–88^.

2 $$R(t)={R_{\hbox{max} }}\left( {1 - {e^{ - {k_a}t}}} \right)$$

where *R(t)* is the reflectivity at time *t*,* R*_*max*_ is the maximum reflectivity at saturation, *k*_*a*_ is the apparent association rate constant, and *t* is the interaction time in seconds. The evolution of association duration and *R*_*max*_ as a function of UA concentration is presented in Fig. 6b. As UA concentration increases, the association duration decreases from approximately 92.8s at 0 mg/dL to about 86.8s for concentrations of 3 mg/dL and above, indicating faster binding kinetics at elevated analyte levels. This trend can be attributed to increased analyte flux toward the sensor surface, which accelerates the establishment of binding equilibrium. Concurrently, the maximum reflectivity increases from 0.45864 at 0 mg/dL to values exceeding 0.60 for UA concentrations between 1 and 5 mg/dL, reflecting enhanced surface occupancy and a stronger plasmonic response. At higher UA concentrations, modest fluctuations in *R*_*max*_ are observed, with a slight reduction at 9 mg/dL followed by recovery at 12 mg/dL. Such behavior likely arises from surface heterogeneity, partial steric hindrance, or local refractive index redistribution as the sensing interface approaches saturation, rather than a loss of binding efficiency^89–91^. The reflectivity increase, calculated from baseline to plateau values of the binding curve, exceeds 1200% across all concentrations, highlighting the strong signal amplification capability of the dual-mode plasmonic architecture. The quantitative parameters summarized in Table 1. Association durations were estimated from the temporal evolution of the reflectivity response, while *R*_*max*_ values correspond to the equilibrium reflectivity reached during the association phase. Together, the angular shift and reflectivity analyses demonstrate concentration-dependent, high-affinity UA binding with accelerated kinetics at higher analyte levels. Notably, optimal analytical performance is observed within the 3–7 mg/dL range, which aligns well with clinically relevant serum UA concentrations, while saturation effects become apparent beyond 9 mg/dL.

**Table 1:** Extracted association phases for different uric acid concentrations.

Uric acid concentration	Start time (s)	End time (s)	Duration (s)	Reflectivity increase (%)	Maximum reflectivity (*R*_max_)
0 mg/dL	0	~ 92.78	~ 92.78	1207% (0.0351 → 0.45864)	0.45864
1 mg/dL	0	~ 89.81	~ 89.81	1449% (0.03891 → 0.60266)	0.60266
3 mg/dL	0	~ 86.83	~ 86.83	1303% (0.04364 → 0.61217)	0.61217
5 mg/dL	0	~ 86.83	~ 86.83	1458% (0.04060 → 0.63257)	0.63257
7 mg/dL	0	~ 86.83	~ 86.83	1364% (0.04167 → 0.61000)	0.61000
9 mg/dL	0	~ 86.83	~ 86.83	1340% (0.03838 → 0.55268)	0.55268
12 mg/Dl	0	~ 86.83	~ 86.83	1491% (0.03959 → 0.62999)	0.62999

### Sensitivity and LOD assessments of the dual-mode sensor

To clarify the contribution of each functional layer and directly demonstrate the advantage of the coupled-mode architecture, the sensing performance was evaluated sequentially for three configurations: bare Au film, Au–APTES–AuNPs, and Au–APTES–AuNPs–rGO. The resonance-angle shift (Δθ_RES_) and the corresponding change in minimum reflectance were recorded as the UA concentration increased from 1 to 12 mg/dL. Angular sensitivity, obtained from the slope of the linear fit in Eq. (3)^92^, reflects changes in plasmon momentum induced by UA-driven refractive-index variation.

3 $$S=\frac{{\Delta {\theta _{RES}}}}{C}$$

The LOD, defined as the lowest UA concentration distinguishable from DI water, was calculated using Eq. (4)^51^, where *SD*_*DI*_ is the standard deviation of ten repeated DI measurements.

4 $$LOD=\frac{{3 \times S{D_{DI}}}}{S}$$

For validation, an empirical comparative LOD was also obtained by identifying the smallest concentration producing an angular shift greater than three times the DI noise level ($\Delta {\theta _{RES}}({C_{\hbox{min} }})>3 \times S{D_{DI}}$).

**Fig. 7:**
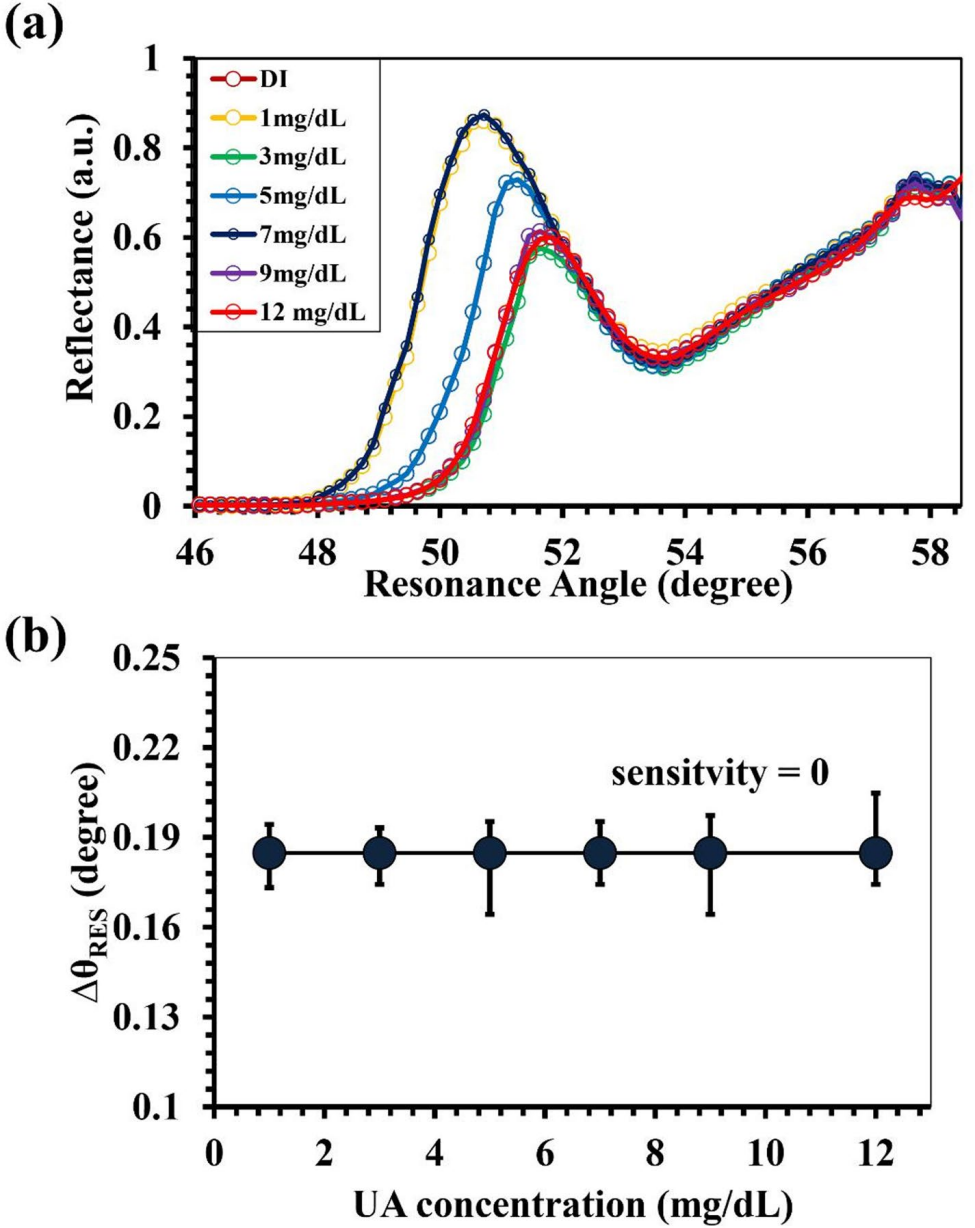
Bare Au control experiment: (**a**) reflectance curves for UA concentrations from 1 to 12 mg/dL, showing negligible resonance-angle displacement; and (**b**) corresponding linearity plot indicating an effectively zero angular sensitivity and LOD.

For the bare Au film, no measurable resonance-angle shift was observed across the tested UA range of 1–12 mg/dL (Fig. 7a). The resonance angle remained essentially constant at 53.8415°, resulting in an effectively negligible sensitivity and LOD, and no meaningful linear correlation (Fig. 7b). This behavior is consistent with previous reports and highlights the limited response of conventional SPR to low-molecular-weight analytes such as UA^51^.

**Fig. 8:**
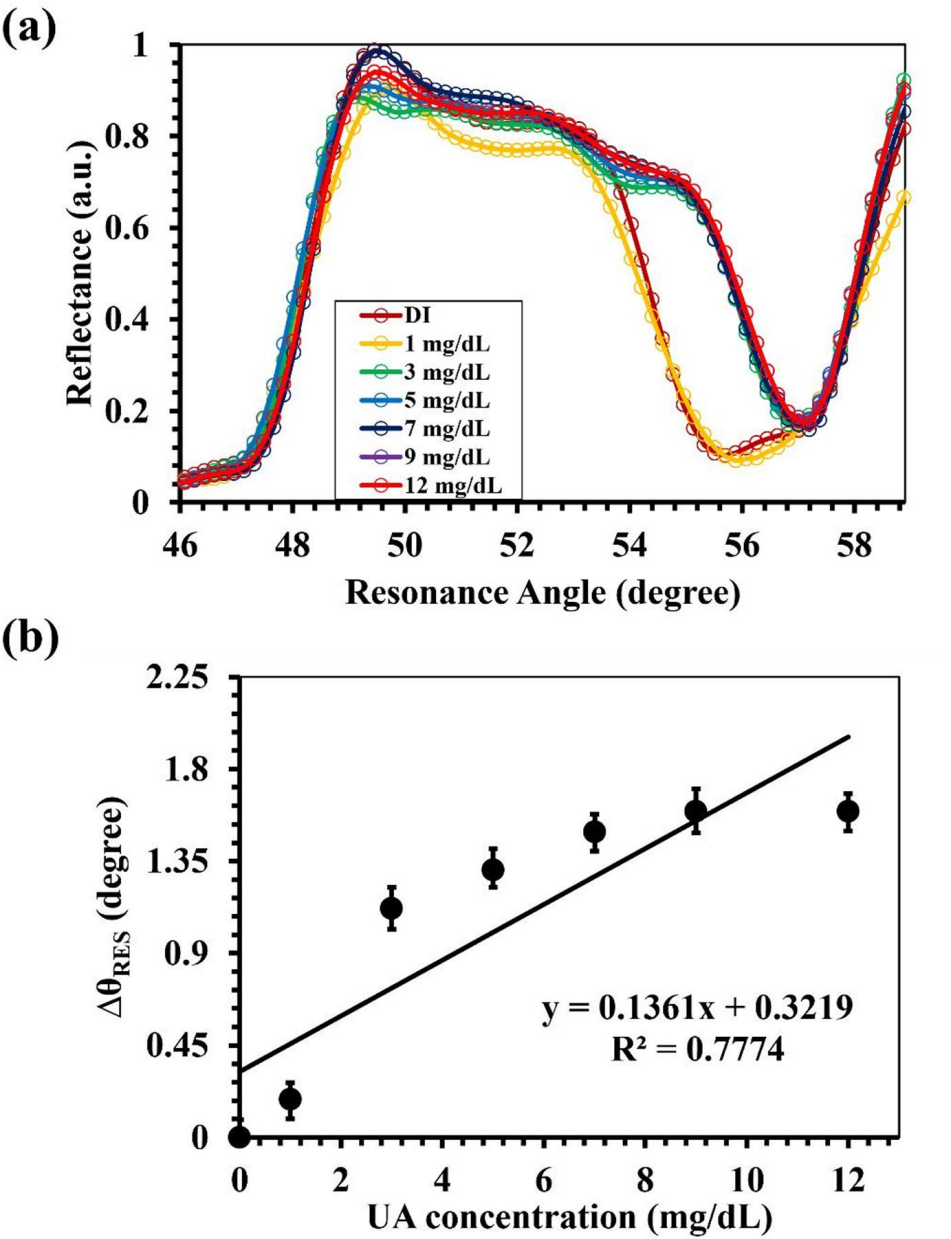
Intermediate Au–APTES–AuNPs control architecture: (**a**) reflectance curves showing concentration-dependent resonance shifts for UA; and (**b**) linearity plot of resonance-angle shift versus UA concentration, demonstrating the onset of LSPR-assisted sensitivity and LOD.

Upon functionalization with APTES and AuNPs immobilization, the sensor exhibited a measurable response. The DI baseline was 55.6983°, and the resonance angle shifted progressively with increasing UA concentration, reaching 57.2913° at 12 mg/dL (Fig. 8a). This intermediate architecture yielded a sensitivity of 0.1361°/(mg/dL), LOD of 0.1905 mg/dL, and linearity of 0.7774 (Fig. 8b), indicating that AuNPs incorporation improves local electromagnetic coupling and enables detectable UA recognition.

**Fig. 9:**
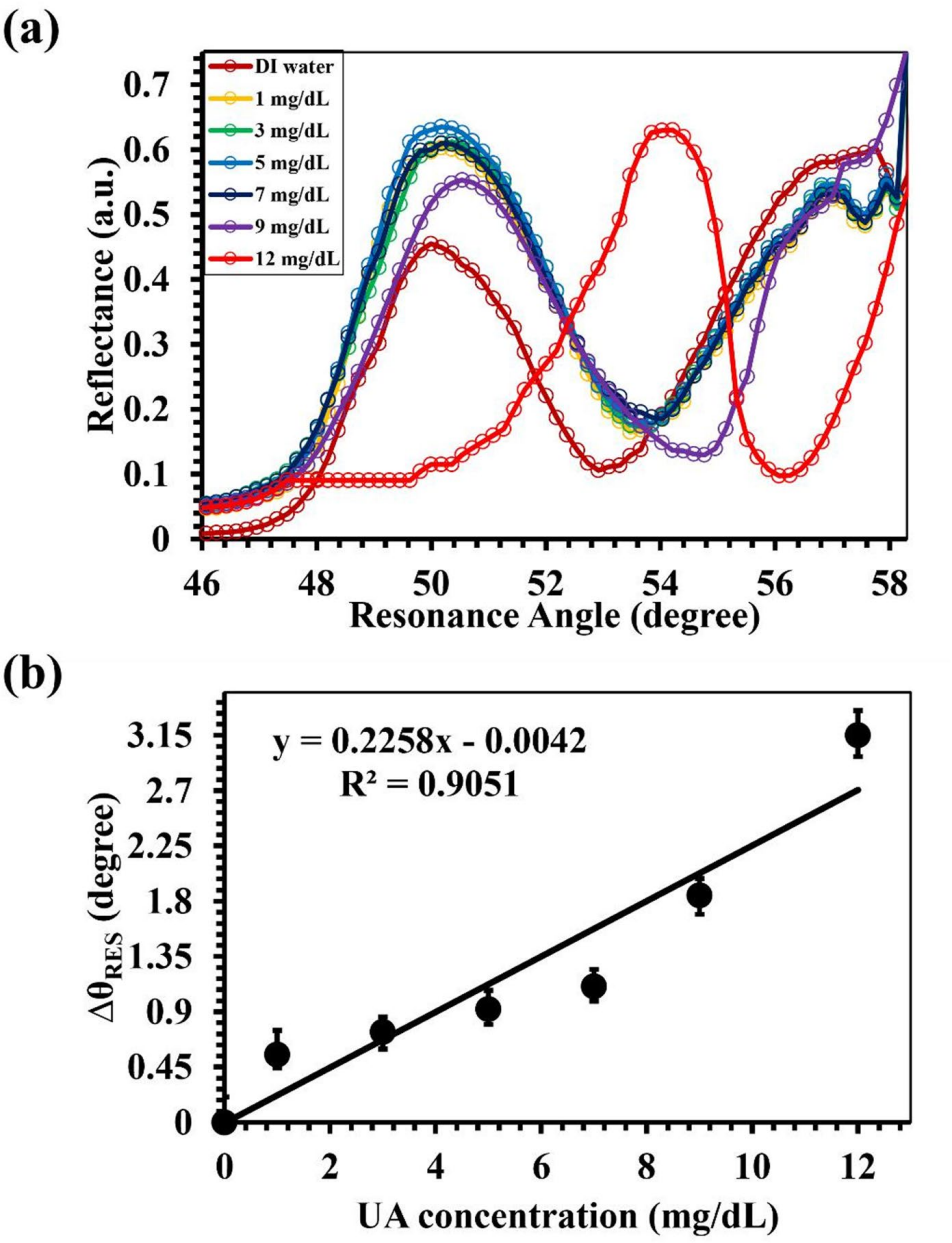
Final Au–APTES–AuNPs–rGO dual-mode sensor: (**a**) reflectance curves showing progressive resonance-angle shifts with increasing UA concentration; and (**b**) linearity plot used to determine angular sensitivity and LOD.

The final Au–APTES–AuNPs–rGO sensor showed the strongest response. The DI baseline was 52.9182°, and the resonance angle increased progressively from 53.4719° at 1 mg/dL to 56.071° at 12 mg/dL (Fig. 9a). The resulting sensitivity of 0.2258°/(mg/dL), LOD of 0.0446 mg/dL, and linearity of 0.9051 indicate that the addition of rGO further enhances surface interaction and signal definition while preserving a sharp resonance dip (Fig. 9b). Slight deviation from linearity at higher concentrations likely reflects partial surface saturation near equilibrium.

Overall, the progressive transition from no measurable response in the bare Au film to moderate sensitivity in the Au–APTES–AuNPs structure and then to the highest sensitivity and best linearity in the Au–APTES–AuNPs–rGO sensor directly demonstrates the stepwise advantage of the dual-mode sensing strategy. These results suggest that AuNPs activate LSPR-assisted field enhancement, while rGO contributes additional adsorption sites and improved interfacial coupling, leading to the most robust overall sensing response.

### Selectivity and interference resistance of the dual-mode plasmonic sensor

The selectivity of the sensor toward UA was evaluated by measuring its response to common interferents, D-cystine, glucose, urea, and creatinine, each prepared at 9 mg/dL and tested under identical conditions. A mixed solution with concentration of 9 mg/dL containing UA mixed with all interferent was also examined to assess competitive binding. Resonance-angle shifts were recorded for all cases, and interference resistance was computed using Eq. (5)^93^.

5 $$\eta =\left| {\frac{{\Delta {\theta _{RE{S_{UA}}}} - \Delta {\theta _{RE{S_{\operatorname{int} erferents}}}}}}{{\Delta {\theta _{RE{S_{UA}}}}}}} \right|$$

**Fig. 10:**
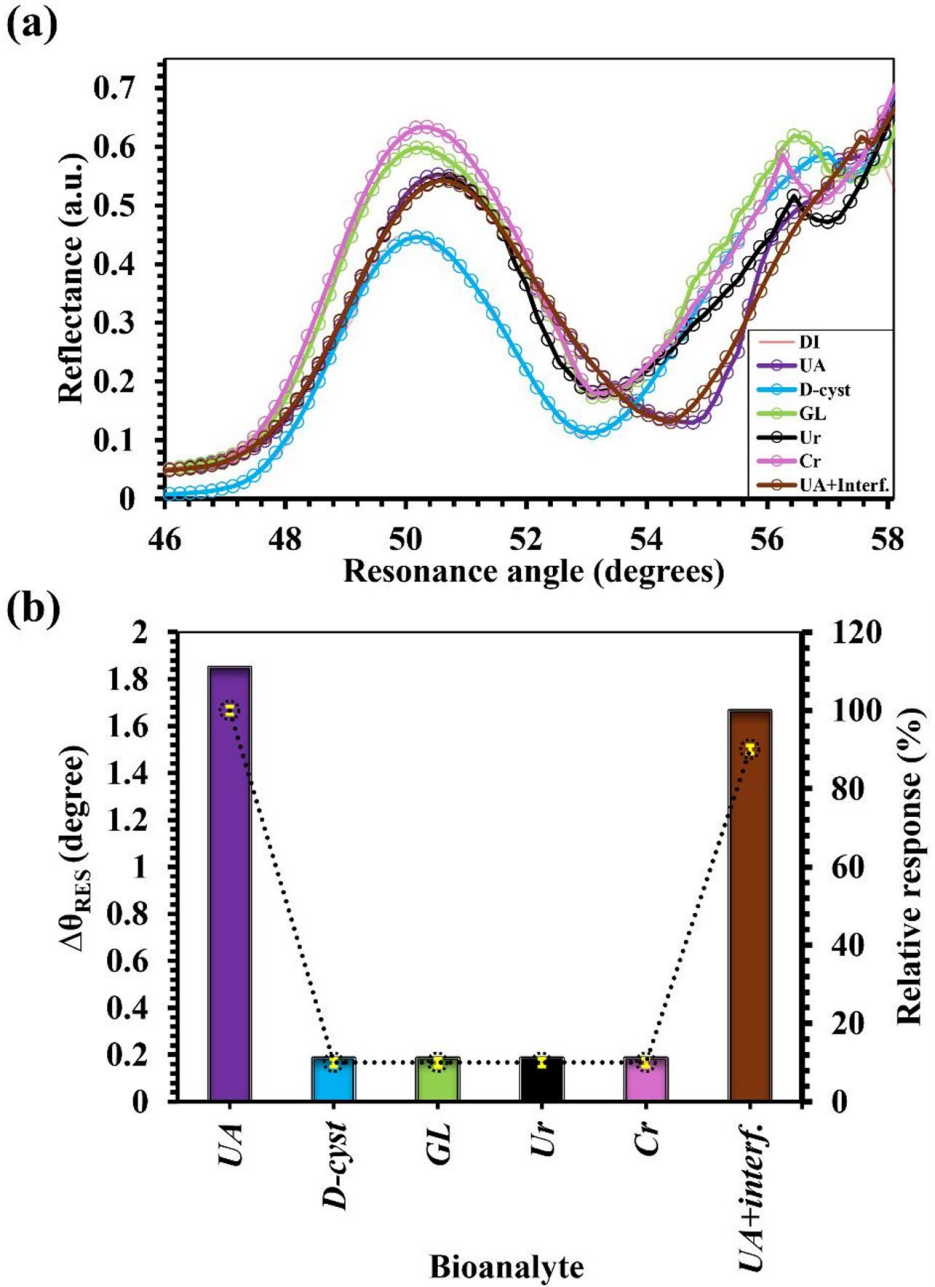
Dual-mode plasmonic sensor’s (**a**) reflectivity plots measuring each analyte, and (**b**) histogram showing resonance angle shifts and relative responses for each analyte.

As shown in Fig. 10a, the resonance angle for UA reached the highest value of 54.7684°, whereas the competing interferents exhibited a lower resonance angle of 53.1025° each. The mixed bioanalyte solution showed a higher resonance angle of 54.5827° relative to the interferents alone, reflecting the presence of UA.

Illustrations in Fig. 10b show that the sensor exhibited a pronounced resonance shift of 1.8502° for 9 mg/dL UA, whereas each interferent produced only 0.1843° at same concentration, indicating that nonspecific interactions contribute just ~ 10% of the UA response. The mixed-analyte solution generated a shift of 1.6645°, corresponding to 90% signal retention relative to pure UA and showing only 10% reduction under simultaneous competition. These results confirm that the dual-mode plasmonic interface intrinsically favors UA binding and maintains high specificity even in the presence of competing bioanalytes.

### Operational stability and reusability of the dual-mode plasmonic sensor

The operational stability of the dual-mode plasmonic sensor was assessed through baseline drift analysis, regeneration performance, thermal tolerance, and long-term storage behaviour.

**Fig. 11:**
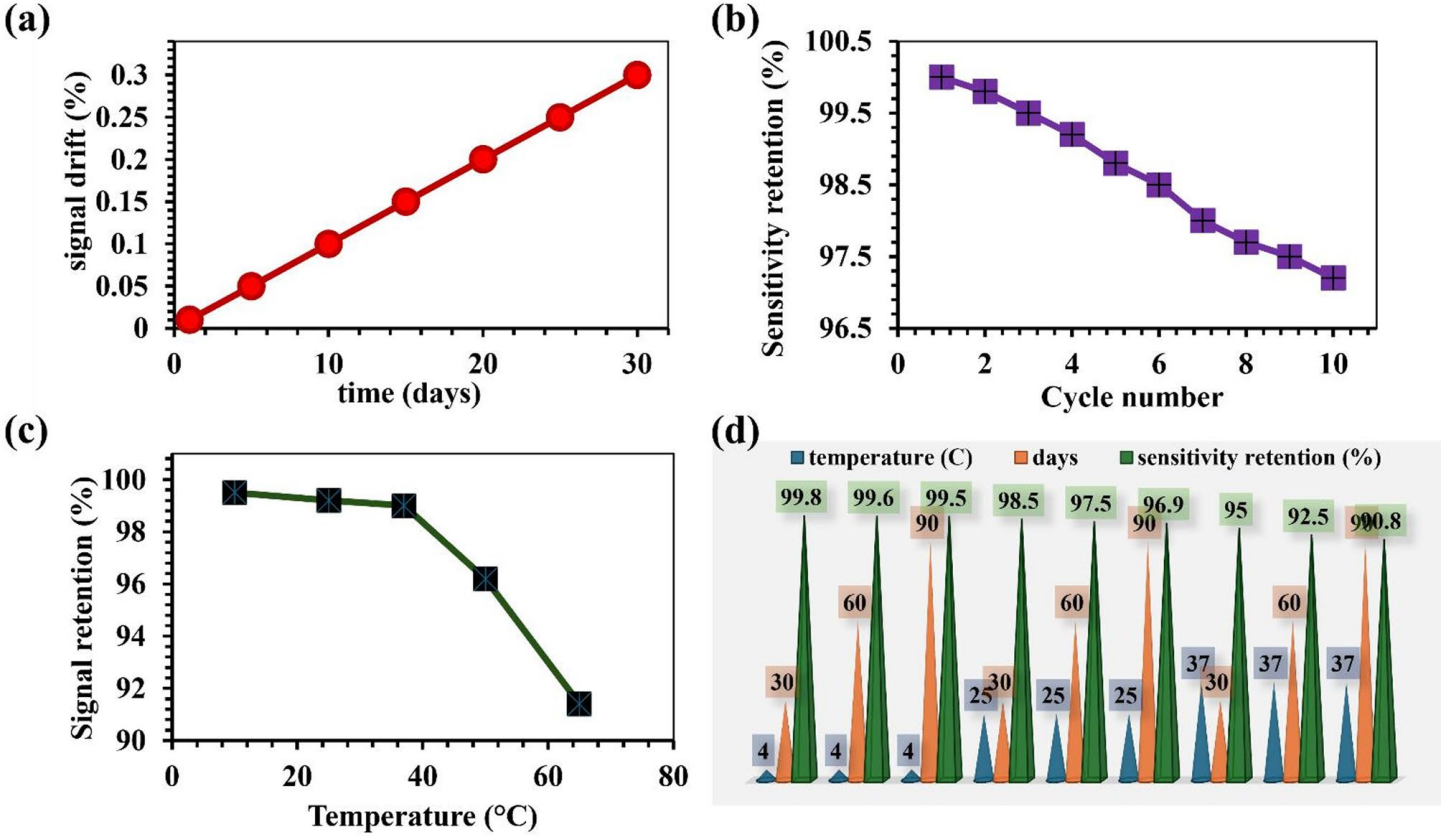
Dual-mode plasmonic sensor stability tests showing (**a**) baseline reflection over 30 days, (**b**) repetitive binding-regeneration (10 cycles), (**c**) thermal performance (signal retention vs. temperature), and (**d**) storage stability.

Baseline stability was monitored by measuring the DI water resonance angle at five-day intervals for 30 days under ambient laboratory conditions (~ 25 °C). As shown in Fig. 11a, the drift remained within ± 0.3%, indicating a stable optical response with minimal influence from environmental fluctuations. The sensor was stored in a covered Petri dish throughout the experiment to reduce contamination.

Reusability was examined by repeatedly exposing the sensor to 3 mg/dL UA, followed by DI rinsing and regeneration using 0.1 M NaOH. This cycle was repeated ten times, with reflectance profiles recorded after each regeneration step. As presented in Fig. 11b, the sensor retained 97.2% of its original sensitivity, demonstrating strong surface robustness, limited ligand degradation, and effective restoration of binding sites. Possible degradation mechanisms during repeated regeneration cycles include gradual hydrolysis of APTES‑derived silane bonds, partial oxidation or etching of the rGO layer during NaOH‑based cleaning, and subtle AuNPs reshaping due to repeated exposure to pH variation and ionic strength changes. These effects may contribute to the slight reduction in signal intensity and binding efficiency observed after 10 cycles. Surface passivation or more gentle regeneration protocols could mitigate such degradation in future implementations.

Thermal stability was evaluated by holding the sensor at 4 °C, 25 °C, and 30 °C for 24 h prior to testing with 1 mg/dL UA. Temperature control was achieved using available laboratory facilities: refrigeration at 4 °C, dual-AC stabilization at 25 °C, and reduced cooling for 30 °C. Figure 11c shows that the sensor retained more than 99% of its response at 4 °C, with small reductions of 3.8% and 8.6% at 25 °C and 30 °C, attributed to partial hydrolysis and oxidative degradation of the surface functional layers, particularly the rGO film.

Long-term storage performance was assessed over 90 days at 4 °C, 25 °C, and 30 °C using sealed containers. At 30-day intervals, the sensor was tested with 1 mg/dL UA after temperature equilibration. As shown in Fig. 11d, sensitivity retention remained exceptionally high at 99.5% at 4 °C, decreased slightly to 96.9% at 25 °C, and dropped to 90.8% at 30 °C, consistent with temperature-accelerated deterioration of surface-bound functional groups.

Collectively, the minimal drift, high regeneration efficiency, and strong thermal and storage stability confirm the robustness of the dual-mode plasmonic architecture. These results demonstrate the suitability of the sensor for continuous analyte monitoring and stable long-term operation in practical diagnostic environments. The stability results were summarised in Table 2.

**Table 2:** Stability test results.

Test/condition	Result/performance metric	Value (%)
Baseline reflectance (30 days at 25 °C)	Signal drift	< 0.3
Repetitive binding-regeneration (10 cycles)	Sensitivity retention	97.2
Thermal performance	Signal retention at 4 °C	> 99
	Signal retention at 25 °C	> 99
	Signal retention at 30 °C	96.2
Storage stability (90 days)	Sensitivity retention at 4 °C	99.5
	Sensitivity retention at 25 °C	96.9
	Sensitivity retention at 30 °C	90.8

### Comparison of developed sensor with existing plasmonic UA sensors

This section compares the dual-mode plasmonic sensor with previously reported non-enzymatic SPR and LSPR platforms for UA detection, as summarised in Table 3. Conventional SPR sensors typically employ Au thin films with dielectric enhancers (ZnO), 0D nanomaterials (GQDs), organosilanes, or uricase, while LSPR configurations leverage AuNPs/Ag nanostructures on optical fibers for localized field intensification. The comparison focuses on state‑of‑the‑art non‑enzymatic plasmonic UA sensors to contextualize the performance of the Au‑APTES‑AuNPs‑rGO platform. For a broader perspective on alternative SPR‑ and LSPR‑based platforms, including enzymatic and non‑biological recognition schemes, see Table 3.

LSPR‑based architectures, such as AuNPs‑coated optical fibers, often exhibit higher nominal sensitivity for small molecules due to strong near‑field confinement and proximity of the sensing volume to the metal surface^94–96^. However, LSPR signals are typically broader and more susceptible to non‑specific adsorption and environmental noise because of limited electromagnetic field penetration depth and reduced spectral resolution^97,98^. In contrast, conventional SPR provides sharper resonance dips and higher refractive‑index specificity, but with lower sensitivity for small‑molecule analytes that are confined to the thin sensing region^84,99^. The dual‑mode Au‑APTES‑AuNPs‑rGO platform developed in this work combines these advantages: the Au film provides a well‑defined propagating SPR with high angular resolution and reference stability, while the AuNPs hotspots and rGO layer enhance near‑field interaction and surface area, thereby improving sensitivity toward small molecules like UA without sacrificing signal definition^100,101^. This synergistic configuration allows the sensor to achieve superior field overlap and maintain a relatively narrow resonance dip while achieving higher angular sensitivity compared with SPR‑only and LSPR‑only counterparts.

Performance hierarchy analysis reveals LSPR fiber sensors peaking at 0.625 nm/(mg/dL) wavelength sensitivity^102^ and 0.125%/(mg/dL) intensity sensitivity^103^, while pure SPR reaches 3.77%/(mg/dL)^51^ or 0.0755°/(mg/dL) enzymatic^52^. The developed Au‑APTES‑AuNPs‑rGO configuration achieves an angular sensitivity of 0.2258°/(mg/dL), which is higher than reported values for enzymatic SPR, Au‑ZnO‑based SPR, and AuNPs‑GQD‑based SPR‑LSPR under comparable conditions, likely due to the high surface area of rGO (~ 500 m²/g) and the combined effect of defect‑mediated UA chemisorption and AuNPs‑driven plasmonic hotspots^45,104,105^.

Binding affinity and selectivity analysis shows that the previously reported Au‑APTES‑GQDs platform exhibits a high binding affinity of 1.6 × 10^8^ (mg/dL)⁻¹, but is more susceptible to non‑specific adsorption^50^. In contrast, the current Au‑APTES‑AuNPs‑rGO configuration achieves a binding affinity of 1451.85 (mg/dL)⁻¹, with a pronounced resonance shift of 1.6645° for UA and relatively low responses to common interferents, indicating good selectivity under the tested conditions, and higher than GQDs-hybrid (1.1757°) and enzymatic (1.1135°) platforms^46,52^. The selectivity, expressed as the resonance‑angle shift ratio between UA and interferents, exceeds 3:1, consistent with the charge‑ and π–π‑based discrimination provided by rGO’s π‑conjugated basal planes and edge‑bound carboxyl groups^43,106^.

Some optical‑fiber LSPR sensors span concentration ranges up to 50 mg/dL^107,108^, but their lower sensitivities can limit resolution at low analyte concentrations. In the present work, the reported LOD of 0.0446 mg/dL lies below 0.05 mg/dL and compares favorably with the MIP‑based SPR platform (LOD 0.025 mg/dL, but over a narrower detection range)^109^, supporting the potential for early detection of hyperuricemia (typically < 3 mg/dL) across the clinically relevant 1–12 mg/dL window (physiological 3–7 mg/dL to gout‑associated > 9 mg/dL).

The non‑enzymatic Au‑APTES‑AuNPs‑rGO design avoids the instability associated with uricase‑based platforms, which retain ~ 88% activity at 40 °C for 30 min but decline to 16–50% after 24–48 h at 50 °C, with complete denaturation above 60 °C^110,111^. The rGO layer provides enhanced electrical conductivity (50–200 S/cm in thin films) and defect‑rich surface sites that complement AuNPs‑driven plasmonic hotspots, rather than relying solely on quantum‑confinement‑based effects in GQDs^112^. It is important to note that the sensitivities tabulated in Table 3 are expressed in different units (e.g., °/(mg/dL), %/ (mg/dL), nm/(mg/dL), mV/(mg/dL)) and under different experimental conditions. Within the set of SPR‑ and SPR‑LSPR‑based UA sensors considered here, the Au‑APTES‑AuNPs‑rGO configuration exhibits relatively high sensitivity, a broad dynamic range, and good selectivity; however, a fully normalized comparison across all sensing modalities (e.g., to refractive‑index change per unit analyte) remains challenging due to these inherent differences in measurement approaches and reporting metrics. Establishing a unified normalization framework could facilitate more consistent cross-platform evaluation in future studies.

The Au–APTES–AuNPs–rGO platform achieves an optimal balance of high sensitivity, low LOD, and strong selectivity without biological fragility, making it a scalable and stable non-enzymatic plasmonic UA sensor for point-of-care diagnostics and continuous monitoring. While the presented SPR–LSPR‑rGO platform focuses on high‑sensitivity, label‑free refractive‑index detection, complementary optical techniques such as surface‑enhanced Raman spectroscopy (SERS) can provide vibrational fingerprint information for more specific molecular identification^113^. In future work, the same AuNPs‑rich nanointerface could be adapted for SERS‑based UA detection, combining the advantages of vibrational specificity with the robustness of the non‑enzymatic rGO‑coated platform.

**Table 3:** Comparison of the developed sensor to other UA plasmonic sensors.

Sensor type	Configuration	Sensitivity	Binding affinity	Detection range	LOD	Selectivity (Δθ_UA_ vs. Interferent)	References
LSPR	Optical fiber/Ag/Si	0.625 nm/(mg/dL)	–	0–15.13 mg/dL	0.0538 mg/dL	~0.8–0.9 (est. ascorbic acid) shows moderate rejection; no explicit interferent data	^102^
SPR	Kretschmann: hybrid Au*–*ZnO	0.0017°/(mg/dL)	–	0–50 mg/dL	10.1 mg/dL	—	^107^
LSPR	Tapered fiber/AuNPs/GO	0.482 nm/(mg/dL)	–	0.17–13.45 mg/dL	3.46 mg/dL	Unquantified ~ 0.95 (est. from linearity)	^114^
SPR	Kretschmann: Uric acid imprinted Poly(HEMA-MAC)- Fe3 + NPs/Au/prism	0.0018%/(mg/dL)	0.0072 (mg/dL)^−1^	0.05 mg/dL – 4 mg/dL	0.025 mg/dL	> 25x (imprinting cavities)	^109^
LSPR	Tapered plastic optical fiber/ZnO	0.025 mV/(mg/dL)	–	0–50 mg/dL	0.56 mg/dL	–	^108^
LSPR	Micro-ball fiber/AuNPs/GO	0.125%/(mg/dL)	–	0.168–15.13 mg/dL	1.103 mg/dL	Unquantified	^103^
LSPR	Tapered fiber/AuNPs/uricase	0.435 nm/(mg/dL)	–	0.168–15.13 mg/dL	2.96 mg/dL	Not explicitly quantified	^115^
SPR	Kretschmann: AU–GQDs/prism	3.77%/(mg/dL)	0.667 (mg/dL)^−1^	1–9 mg/dL	4.74 mg/dL	–	^51^
SPR	Kretschmann: Au–APTES–11-MUA–EDC/NHS–uricase/prism	0.0755°/(mg/dL)	298.83 (mg/dL)^−1^	0.5–12 mg/dL	0.095 mg/dL	1.1135° (Enzyme-dominated; pH-sensitive)	^52^
SPR	Kretschmann: Au–APTES–GQDs/prism	0.0355°/(mg/dL)	1.6 × 10^8^ (mg/dL)^−1^	0.5–12 mg/dL	0.2 mg/dL	–	^50^
SPR-LSPR	Kretschmann: Au–APTES*–*AuNPs*–*GQDs	0.1828°/(mg/dL)	–	1– 9 mg/dL	0.1658 mg/dL	1.1757° (Hybrid LSPR boosts field but GQDs limits conductivity)	^46^
SPR-LSPR	Kretschmann: Au–APTES*–*AuNPs*–*rGO	0.2258°/(mg/dL	1451.85 (mg/dL)^−^¹	1– 12 mg/dL	0.0446 mg/dL	1.6645°	This work

More recently, photonic structures such as surface‑lattice resonances (SLR), Fano‑resonant metasurfaces, and bound‑states‑in‑the‑continuum (BIC) modes have been explored for high‑Q, narrow‑linewidth sensing^116–120^. These architectures can achieve extremely high Q‑factors and narrow linewidths, enabling ultra‑high theoretical sensitivity per unit refractive‑index change^121–123^. However, their practical implementation often requires precise nanofabrication, periodic arrays, or complex dielectric environments, which can limit robustness and ease of integration into point‑of‑care devices^124,125^. In contrast, the Au‑APTES‑AuNPs‑rGO SPR‑LSPR platform uses a planar prism‑coupled configuration with randomly distributed AuNPs and solution‑processable rGO, offering a balance between performance, manufacturability, and operational stability. While the current work does not directly implement SLR, Fano, or BIC modes, it is conceivable that the same hybrid matrix could be integrated into such architectures in future studies to combine high‑Q modes with non‑enzymatic robustness.

## Conclusion

This work presents a non-enzymatic dual-mode plasmonic sensor that achieves unparalleled sensitivity, selectivity, and operational robustness for UA detection. By integrating gold thin films with APTES-functionalized AuNPs and rGO, the sensor harnesses synergistic plasmonic coupling to unify the far-field detection advantage of SPR with the near-field amplification of LSPR. This nanostructured matrix significantly improves local electromagnetic field confinement and molecular recognition capability. In operation, the sensor exhibited a high sensitivity of 0.2258°/(mg/dL), a low detection limit of 0.0446 mg/dL, and a high binding affinity of 1451.85 (mg/dL)⁻¹, outperforming both conventional SPR and LSPR platforms. Its high specificity was validated through selective detection in the presence of interferents, with a 1.6645° resonance shift and 10% interference resistance, highlighting its reliability for clinical diagnostics. The sensor maintained < 0.3% signal drift over 30 days, retained 97.2% sensitivity after 10 regeneration cycles, and sustained > 90% thermal performance up to 30 °C. Storage stability was equally impressive, with > 96.9% sensitivity retention after 90 days. Crucially, the sensor’s broad dynamic range (1–12 mg/dL) optimally spans physiological (3–7 mg/dL) to pathological (> 9 mg/dL) concentrations, making it ideally suited for real-time monitoring and POCT applications. Unlike enzymatic systems, this non-enzymatic design eliminates issues related to enzyme degradation and environmental instability, while also offering ease of fabrication and long shelf life. Beyond UA detection, the platform’s modular architecture allows for adaptation to a wide array of bioanalytes through tailored bioreceptor and nanomaterial integration. This work represents a promising non-enzymatic plasmonic UA sensor architecture with strong performance for real-time diagnostics, contributing to the advancement of next-generation biomedical plasmonics. While the platform shows clear potential for clinical and point-of-care use, validation in real biological matrices such as serum or saliva remains an important next step, as these complex media may introduce fouling and background effects. Future efforts will therefore focus on testing in relevant biological samples, improving surface passivation strategies, as well as advancing multiplexed detection, microfluidic integration, and miniaturization for portable applications.

## Data Availability

All data required to reproduce these findings are included into the paper.
